# Exploring Individuals’ Views and Feedback on a Nutritional Screening Mobile App: Qualitative Focus Group Study

**DOI:** 10.2196/63680

**Published:** 2024-12-18

**Authors:** Debra Jones, Anne Marie Sowerbutts, Sorrel Burden

**Affiliations:** 1 School of Health Sciences University of Manchester Manchester United Kingdom

**Keywords:** malnutrition, malnutrition risk, malnutrition screening, MUST, mobile application, mHealth app, malnutrition universal screening tool

## Abstract

**Background:**

Malnutrition is a major global health challenge. Worldwide, approximately 390 million adults are underweight, while 2.5 billion are overweight. The Malnutrition Universal Screening Tool (MUST) has been implemented successfully in the United Kingdom to assess the nutritional status of patients in health care settings. Currently, MUST is available as a web-based tool or as a paper-based version, However, the paper tool can lead to calculation errors, and web-based tools require internet access, limiting use in some communities. The MUST app uses clear and simple navigation and processes information precisely, so could potentially improve the accuracy and accessibility of malnutrition screening for health care professionals (HCP) in all settings.

**Objective:**

This study aimed to explore the views of HCPs on the content, functionality, and usability of a newly developed mobile app for MUST.

**Methods:**

We performed a qualitative study using deductive and inductive framework analysis. A series of online focus groups (~1 hour each) were conducted, exploring potential users’ views on the app’s content design, functionality, and usefulness, which was set in demonstration mode and not available for direct use with patients. Each focus group used a semistructured approach and predefined topic guide. Participants were recruited consecutively and United Kingdom–wide using advertisements through emails, newsletters, and on social media across appropriate local and national networks. Participants had the opportunity to look at the app on their phones before giving feedback and an on-screen demonstration of the app was provided during the focus group. Data were analyzed using deductive and inductive framework analysis.

**Results:**

In total, 8 online focus groups were conducted between August 2022 and January 2023. Participants (n=32) were dietetic and nutrition HCPs or educators with experience in using MUST in clinical or community settings. Data analysis revealed three broad themes: (1) improving the app for better use in practice, (2) user experience of design, and (3) barriers and facilitators in different settings. Overall feedback for the app was positive with potential users considering it to be very useful for improving routine and accurate screening, particularly in the community, and mainly because of the automatic calculation feature, which may help with improving discrepancies. Participants generally considered the app to be for professional use only, stating that patients may find it too clinical or technical. Participants also made suggestions for app sustainability and improvements, such as incentives to complete the demographics section or the option to skip questions, and the addition of more subjective measures and instructions on measuring ulna length.

**Conclusions:**

The MUST app was positively evaluated by potential users, who reported it was user-friendly and an accessible way to screen for malnutrition risk, whilst improving the accuracy of screening and availability in community settings.

## Introduction

Malnutrition is an imbalance of a person’s energy intake or intake of certain nutrients, causing undernutrition, vitamin or mineral deficiencies, or obesity; and contributing to diet-related diseases [1,2]. Malnutrition is a major global health challenge, with unhealthy diets causing more adult deaths and disabilities than smoking and alcohol [3]. Worldwide, around 390 million adults are underweight, while 2.5 billion are overweight, with 890 million of these living with obesity [2]. In the United Kingdom, malnutrition is a common health care problem [4,5], affecting around 2.65 million people and costing the National Health Service £19.6 billion (US $24.6 billion) every year [4,6,7]. It is also estimated that malnutrition affects 16,719 per 100,000 people who are over 50 years old in the United Kingdom [8], with many of these being community-based [5,9]. Nutritional screening, the first key stage in addressing malnutrition, can vary across health care settings, leading to underrecognition and undertreatment [6,10].

In United Kingdom secondary care, screening to identify malnutrition in adults has been implemented successfully with the use of the Malnutrition Universal Screening Tool (MUST), which is a commonly used tool in United Kingdom hospitals [11]. Similarly, surveys conducted between 2007 and 2011, including 474 UK care homes, reported that the majority (96% in 2011) used MUST to screen for malnutrition [12]. In 2022 a national survey of screening in hospitals with MUST indicated that 44% of in-patients were at medium to high risk of malnutrition [13]. Data from the same survey also stated that the prevalence of malnutrition risk in all settings was 45%, with the highest prevalence being in those screened at home (55%) or screened-in care homes (55%) [13]. This is an increase in figures reported from 2007 to 2011 surveys, which stated that 35% of care home residents were deemed to be at risk [12]. This emphasizes the necessity for greater implementation of nutritional screening in care homes and community settings in the United Kingdom, which has previously been highlighted as a research priority [14].

In Europe the prevalence of malnutrition risk varies widely between countries and across health care settings, potentially a result of the variation of screening tools being used [15], and this is an issue that extends to other countries outside of Europe [16]. As in the United Kingdom, the risk of malnutrition poses a significant problem across Europe, with data from 20,000 hospitalized patients in 25 European countries indicating that 27% are “at nutritional risk” [17]. In addition, pooled prevalence rates of people at risk of malnutrition from 583,972 older adults across 24 European countries were 28% in hospitals, 17.5% in residential care, and 8.5% in community settings [15].

Despite malnutrition being a major challenge in the United Kingdom, it is encouraging that MUST is now widely used in hospitals and validated for community and residential care settings [18]. Developed by the British Association for Parenteral and Enteral Nutrition (BAPEN) and recommended by The National Institute for Health and Care Excellence (NICE), MUST has been extensively validated for its validity and reliability [1,19]. MUST incorporate BMI, percentage weight loss, and “acute disease effect” to assess nutritional risk [1]. The tool is available both as a web-based tool [20] and in paper format for health care professionals (HCPs) [21], with a self-screening version for the public launched in 2015 [22]. However, the paper tool can lead to calculation errors, and web-based tools require internet access, limiting their use in some communities. A mobile app of MUST would provide a clear and simple user interface with information processed precisely both on- and offline, potentially providing HCPs with a more accurate and accessible method for nutritional screening. The availability of an app in community settings, including community health and walk-in centers where internet access may be limited, would align with the new National Health Service (NHS) plans, where key and digitally enabled services will move to primary care [23].

The purpose of the MUST app is to readily identify people within all health care settings who are at risk of malnutrition and signpost to an appropriate plan of action. The aim of this project was to evaluate the content, design, functionality, and usefulness of the MUST digital mobile app, before its official release, to inform further app development that ensures a high level of usability in relation to content, architecture, and user interface. The app is currently in demonstration mode, only accessible with permission from software engineers and is not yet available for use with patients.

## Methods

### Design

This is a qualitative study using deductive and inductive framework analysis [24,25], designed to capture individuals’ experiences, views, and feedback on the MUST app. Online focus groups with potential users of the MUST mobile app were conducted between August 2022 and January 2023. The subsequent report of the study follows the COREQ (Consolidated Criteria for Reporting Qualitative Research; Multimedia Appendix 1) [26].

### Ethical Considerations

We used the University of Manchester ethics assessment flowchart to formally obtain an ethical exemption from the formal committee approval system. The work was with professionals and asked questions strictly within their professional remit, relating to the content and usefulness of a new nutritional screening app, which is currently set in demonstration mode and not available for use with patients. A letter of exemption was provided and is included with this submission. Participants were provided with a participant information sheet a few days before the focus groups to allow time to read and ask any questions. All those participating in the focus groups signed and returned the consent form before attending the focus group. All transcripts of the focus groups were anonymized by the removal of names and any identifiable information, and all recordings were deleted immediately after transcription. No compensation was provided for participation in the focus groups. No images were used.

### Development of the MUST App

**Figure 1:**
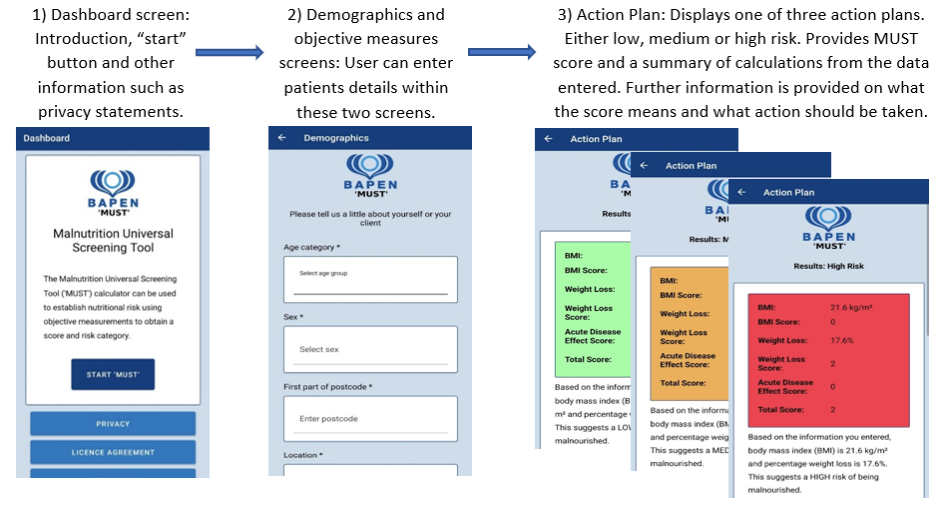
Overview and visuals of the MUST mobile app screens presented to potential professional users during 1-hour online focus groups for feedback. Reproduced here with the kind permission of BAPEN (British Association for Parenteral and Enteral Nutrition) [20]. Copyright BAPEN 2012 (license number LIC2206). MUST: Malnutrition Universal Screening Tool.

The MUST app was developed over several iterations by research software engineers at the University of Manchester with the first iteration based on the current web-based and paper-based MUST tools. Ongoing development was guided by the study researchers (DJ and SB). The first accessible, demonstration version of the app was reviewed by DJ and SB, who provided feedback for subsequent development and modifications until a demonstration version of the app was considered ready for external review by potential users within focus groups (Refer to Figure 1).

### Participants

Participants were HCPs or educators with a dietetic or nutritional background who had knowledge or experience of using MUST in a clinical or community setting. Participants were recruited consecutively and United Kingdom–wide using advertisements through emails, newsletters, and on social media across several local and national networks including BAPEN, Malnutrition Action Group, British Dietetic Association, Greater Manchester Nutrition and Hydration Programme, Age United Kingdom Salford, Salford Clinical Commissioning Group and National Nutrition Nurses Group. Those who agreed to participate in the online focus groups were provided with a participant information sheet and returned a signed consent form before attending the focus group. A few days before the start of each focus group, participants were sent a link to allow them to download and access the demonstration version of the app. Participants were asked to look at the app and run through the tool using mock answers to gain an understanding of how the app worked. In total, 33 professionals initially expressed interest and were sent an information sheet. Of these all were deemed eligible and were included in the focus groups; however, one was not available to attend on the day. Data collection and analysis proceeded in parallel and as such recruitment continued until no new themes or insights were emerging and the range of views was considered sufficient to have reached data saturation.

### Data Collection

During the online focus groups, participants were asked to provide feedback on the content, design, functionality, and usefulness of the mobile app. As well as the MUST app being directly available on participant phones, an on-screen demonstration was presented at the start of each focus group. The focus groups were led by one interviewer (DJ). Each focus group ran for approximately 1 hour and participants were asked to provide feedback on the app by the interviewer who conducted the focus groups using a semistructured approach guided by a predefined topic guide (Multimedia Appendix 2), which had been peer-reviewed before use. A second interviewer (AMS) supported each focus group to help keep the conversation on time and meaningful. No other nonparticipants were present for the focus groups. Field notes were kept during the course of each focus group to aid with follow-up on important concepts and to explore for further information. A summary of the main points was relayed during each focus group to confirm the accuracy of the points raised. Each focus group was conducted once with no repeats being required. All focus groups were conducted over Zoom (teleconferencing software; Zoom Video Communications) and both visual and audio communications were recorded through Zoom’s recording functionality, although visual recordings were only used to aid transcription and were not used in the analysis. Recordings were later transcribed verbatim and then permanently deleted. All transcripts were anonymized by the removal of names and any identifiable information. Transcripts were checked against focus group notes but were not reviewed by participants themselves.

### Data Analysis

Data were managed using NVivo (version 12; QSR International) [27] and analyzed using Framework analysis [24,25]. In alignment with Framework analysis, the thematic framework was developed both inductively from the themes emerging from the focus group transcripts and deductively using the research questions and topic guide [25]. The 5-step process of the Framework analysis involves (1) familiarization, (2) developing a thematic framework, (3) indexing, (4) charting, and (5) data mapping and interpretation. One researcher (DJ) listened to the recorded focus groups to start the process of familiarization and then transcribed verbatim. The same researcher then read and reread the transcripts to become familiar with them. The thematic framework (Multimedia Appendix 3) was applied to the focus groups and then summarized into charts. The charts were discussed and agreed upon between DJ, AMS, and SB. Finally, data mapping and interpretation were discussed and agreed upon between DJ, AMS, and SB. Where quotations are used, only a professional role is noted to preserve confidentiality. Relevant quotes for each subtheme can be found in (Multimedia Appendix 4).

### Rigor

Rigor was introduced by DJ keeping a reflective diary during the study and field notes from the focus groups. Rigor was also introduced by DJ, AMS, and SB discussing and agreeing on the charting, data mapping, and interpretation. The positionality of the researchers was incorporated into the reflection in the understanding and interpretation of the data. All researchers were female and either dietitians or nutritionists with a PhD or MSc and mixed experiences in clinical practice and research in the NHS, community, and acute care settings.

## Results

### Focus Groups

**Figure 2:**
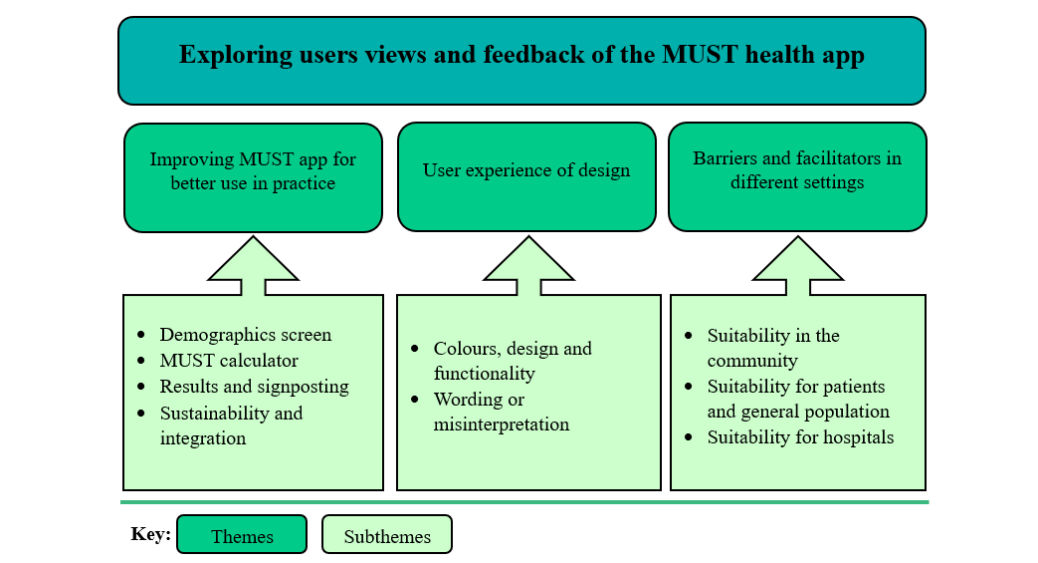
Qualitative themes and subthemes were identified from dietetic and nutrition specialists’ feedback on the newly developed MUST application during 1-hour focus groups. MUST: Malnutrition Universal Screening Tool.

In total 8 focus groups were conducted between August 2022 and January 2023 and 32 dietetic or nutrition specialists provided MUST app feedback. Participants included 17 dietitians, 8 nutrition nurses, 5 educators of nutrition and dietetics, and 2 community dietitians (CDt). The feedback received, which is discussed in full below, fell into three broad themes: (1) improving the app for better use in practice, (2) user experience of design, and (3) barriers and facilitators in different settings (refer to Figure 2). Some of the suggestions for improving the app could be implemented immediately, whilst others were more complex and for possible future implementation. A summary of the suggested immediate and future improvements are displayed in (Table 1) and a summary of the perceived barriers and facilitators for the use of the app in different settings are displayed in (Table 2).

**Table 1:** Summary of recommended immediate and future enhancements for the MUST (Malnutrition Universal Screening Tool) mobile application based on qualitative feedback from dietetic and nutrition specialists during 1-hour focus groups.

Immediate changes		Future changes
**Dashboard and admin**		
	Above the start button add an extra sentence: “This app is intended for the use of HCP^a^ and SCP^b^”	Include a video of how to use the app.
	Under “What will you use the data for”: Change to “nutritional status of people within primary and secondary health care settings” (not “people with long covid”)	Extra button on the dashboard describing MUST^c^, who the app is for, and how to use it
	Under “Who will you share the data with”: change “Manchester NHS^d^ Foundation Trust” to “HCPs directly associated with the project and working within the care setting where data is collected”	Ability to store data, that is–clinicians’ own profile with all client’s entries saved. Or create patient profiles.
	Fix problems occurring on some devices, where parts of the app are not visible in dark mode.	When the app launches ask if a user is HCP or patient and tailor pages to fit
	—^e^	Work with hospitals and BAPEN^f^ to make the app available to download to hospital handheld devices.
**Demographics**		
	Option to hide demographics page for use in the clinical setting? Or this may be completely removed and only used for research purposes.	Move the demographics page to the end so the app opens with the measurements page.
	Change wording at the top to: “Please tell us a little about the person being assessed”	Preselection at the start of what you want to do and the ability to just do MUST, the ability to have settings for research or clinical practice
	Option to say “do not know” for disease	—
	Postcode: Change wording from “First part of postcode” to “First part of postcode for current location*”	—
**MUST calculator**		
	Under the estimate height section, next to the word ulna add “(forearm)”	Additional estimation button that will allow an estimation of BMI using MUAC^g^ or other surrogate measures
	Under the ulna (1) add text and hyperlink: “see MUST explanatory booklet for how to measure ulna” and add a hyperlink to the booklet.	On the front page have the option to select if you have weight and height or not. Then taken down the subjective or objective route.
	Option to enter ulna length in inches as well as cm (with toggle button) plus provide estimated height result in ft/in as well as cm (with toggle button)	Add in step 1, step 2, etc so it looks like a paper-based version.
	Arrows (<,>) should be explained as this is not understood by some HCP	Estimated height can be a significant driver of inaccuracy in the total MUST score if just self-reported and not measured. Highlight the importance of measuring correctly (measure 3 times)
	ADE^h^ explanation is important. Information should be on screen without pressing “” and highlight “AND” to show that to answer yes it must be both acute illness and no nutritional intake, not just one or the other.	Include information and the option to enter information relating to amputation and edema which can influence weight.
	Highlight that previous weight 3-6 months ago should be the biggest possible difference in weight during that time.	—
**Results and signposting**		
	Implement local policy should be highlighted in bold to emphasize that this is the first point of call	Tailored to the NHS site that’s using it. For example, in some hospitals, it may not be possible for a dietitian to see all patients with a score of 2 due to prioritization.
	Give the definition or cutoffs of obesity	Add recommendations about follow-up according to different health care settings, for example how often you should review the patient.
	In 1st paragraph font of the result “Low risk” or “medium risk” or “high risk” should be in its corresponding color (green or orange or red)	Ability to view the data that was entered for calculating the score
	Under “treat” include “Contact GP^i^” and “implement appropriate care pathway”	—
	Option to reset or restart or clear. For ease of entering multiple patients one after another	—

^a^HCP: health care professional.

^b^SCP: social care professional.

^c^MUST: Malnutrition Universal Screening Tool.

^d^NHS: National Health Service.

^e^Not applicable.

^f^BAPEN: British Association of Parenteral and Enteral Nutrition.

^g^MUAC: Mid-upper arm circumference.

^h^ADE: acute disease effect.

^i^GP: General Practitioners.

**Table 2:** Summary of barriers and facilitators for use of the MUST^a^ mobile app as perceived by dietetic and nutrition specialists during 1-hour focus groups.

Barriers			Facilitators
**Community**			
	Might be difficult to record down MUST details and score if no option to store and access them later	Quick and easy for community staff to use	
	Training should be given to all staff before use	Better than the paper-based version and more accessible than the web-based version	
	—^b^	Would fit in well with the MUST training currently offered to care home staff	
	—	Allows for accurate calculations so reducing error and improving referrals	
**Patients and the general population**			
	May not know all information for MUST	Patients and family members may want to self-screen and monitor change or risk	
	Wording, especially on the results screen, is targeted at HCP^c^	Easy access especially if a QR code is provided	
	Use by the general public may lead to inaccuracies	—	
	The app does not have enough instructions available for the general public in terms of accurately measuring height and weight	—	
	The word “malnutrition” may confuse people or put them off	—	
	Some people may have difficulties downloading an app	—	
**Hospitals**			
	Not enough dietitians for MUST referrals	Hospital electronic systems can be difficult to use, and the MUST app is simple. Seen as a “proper” or “validated” app as backed by BAPEN^d^ with BAPEN branding	
	MUST is too generic and can miss patients	The app could be useful for audits	
	Many hospitals’ electronic systems already have a version of MUST built in	The app could help with meeting NICE^e^ standards for malnutrition screening	
	Currently unable to store data or track patients	—	

^a^MUST: Malnutrition Universal Screening Tool.

^b^Not applicable.

^c^HCP: health care professionals.

^d^BAPEN: British Association for Parenteral and Enteral Nutrition.

^e^NICE: National Institute for Health and Care Excellence.

### Improving MUST App for Better Use in Practice

#### Demographics Screen

Demographic questions are asked on the first screen of the app before MUST questions. This is for research purposes and for potential future development of the app, which could include national and geographical monitoring of the screening of malnutrition risk. However, most focus group participants expressed concern about these questions, suggesting that these may deter users or cause confusion:

…people who self-screen are going to open it and go ooh wait a sec I’m suddenly getting asked all these questions about postcode and demographics and actually…I don’t know.

Dietitian11

There was also concern about whether people would be comfortable sharing personal details and if they would question the need for collecting them. In addition, participants reported that users would favor convenience and speed and so would prefer not to complete the demographic questions. Finally, it was suggested that the option could be given to the user to turn off the demographic questions and just go straight into the MUST tool. Concern was also raised about the diagnosis question within the demographics screen. One participant stated that users may find it difficult to complete due to either not knowing their diagnosis or having multiple conditions. One participant also commented that they expected results to be tailored to the diagnosis selected, which is not something the app currently offers. Further feedback was provided in relation to the postcode question within the demographics screen, with participants stating it was not clear if this was the home postcode or postcode of the current location.

#### MUST Calculator

For reporting weight and height, participants generally agreed that this could be measured in several different ways and not all were accurate. Therefore, it was thought that a way to note how weight and height had been measured or recorded was important:

…could you have a drop-down box to say whether it's estimated, actual or MAC (mid arm circumference).

CDt1

Similarly, it was suggested that accuracies in MUST calculations would be greatly improved by extra options or explanations around the reporting of weight 3-6 months ago, which can be problematic if no previous weight has been recorded or if a patient can not accurately provide this information. One of the questions required for the MUST calculation is the “acute disease effect (ADE) score” and participants stated that there were often difficulties or misunderstanding around this. To avoid common misconceptions or inaccuracies, including that ADE is not applicable in the community and is only a requirement of hospital patients, it was suggested that information connected with this question could be made clearer on the app. Participants also noted that as with the paper-based and web-based MUST, there is no way to record if patients have additional conditions that could affect their weight, which then affects MUST score, such as edema or amputation. Even though this has not been accounted for in the past it was suggested that the option to at least record this information would help improve accuracies in MUST screening. Subjective, surrogate, or proxy measures were discussed thoroughly in most groups, with many stating that they often used subjective measures as it is not always possible to measure a patient:

…sometimes we use the mid upper arm circumference or subjective evidence of weight loss.

Dietitian5

One participant also highlighted that surrogate measures are particularly useful in primary care. Many participants suspected that HCPs would not necessarily have all the information required to complete MUST so having other options would help with usability and completion. Currently, the only proxy measure included in the app is the ulna length, which can be used to estimate height. Participants approved of this but suggested that more information was needed to show how to measure the ulna:

…if you want people to use ulna height you need to have a link to show people how to measure ulna height

Dietitian8

One participant suggested that visual pictures for how to measure substitute measurements, including ulna, would be useful. However, it was also noted that alternative and subjective measures may affect the MUST score and influence the tool’s validity and reliability.

#### Results and Signposting

On the results screen it was noted that there was no quick way of clearing the data and starting again:

…when you get to the end of the results page you’re having to go back twice to restart or to do it again.

Dietitian2

Participants also suggested that it would be useful to give the cutoffs for obesity next to the displayed BMI results. It was also highlighted that the results page does not display the MUST information that has been entered and suggested it might be useful to add this. Participants were also keen on the idea of signposting out further information so that users could find further detailed information or links out to other useful resources. It was suggested that a link should be included to the BAPEN explanatory booklet:

I know at the bottom you signpost to the booklet. Could there be a link, I’m just thinking in practice where can people find information.

Educator of nutrition and dietetics2

Another participant stated that linking to other relevant information might be useful, particularly to nondietetic users. It was also highlighted that the MUST explanatory booklet provides helpful pictures for the explanation of MUST, something that would be difficult to integrate into the app.

#### Sustainability and Integration

For continual improvements, it is essential that the app is supported moving forward to keep it up-to-date and functioning correctly. Participants were keen to see the app well supported and stated that linking the app to local care plans and targets would help to incentivize use and so generate income:

In the future, is there going to be a user license agreement. As in, this is our Greater Manchester high-risk care plan. Or thinking about how does this link into primary care? How is going to make the life of the busy GP actually bother to think about nutrition screening when someone is in front of them?

Dietitian1

The discussion generated from this related to how easy access to screening through the app as well as demonstrating how it can help GPs meet weight and BMI record keeping would encourage the use of the app in primary care and potentially increase screening. Furthermore, participants thought that collaboration with BAPEN and using the app on a wider scale nationally would help to support and sustain it. Participants who were hospital-based dietitians highlighted that many of the hospital electronic record systems already have a version of MUST built in and this often links from the patients’ main observations. As such it was thought that secondary care would not need an app for MUST:

…we’ve just launched this new system called [electronic system name] and it would just be the difficulty of flicking between the two systems

Dietitian14

However, some commented that the hospital system was not easy to use.

### User Experience of Design

#### Colors, Design, and Functionality

Overall feedback for the design of the app was very positive and participants reported that the colors and branding enhanced the visual appearance and display:

First impressions are that it looks really clear, nice coloring, nice and bold and fresh.

Dietitian7

Participants also liked the traffic light color codes used for the results screen. In general, participants were happy with the layout of the app. One participant liked that the MUST questions were asked in the same order as the original:

So, if you were doing MUST in clinical practice, you would expect it to come in that order and you’d probably have that information ready to input. And the boxes came up in that order so yeah that was great

Dietitian12

However, another participant said this could be improved by adding “step numbers” like on the paper version of MUST. Positive feedback was given for the layout of the results page, which was thought to be clear and allow for understanding of the calculation. Feedback for functionality and usability was also very positive with participants commenting on how quick it was to complete and the ease of use with dropdown boxes:

…very self-explanatory, very straightforward. I like the fact that it has dropdown choice boxes, so it’s quite quick to do rather than having to input all the information sort of by hand almost. From the point of view of busy nursing staff that’s good.

Nutrition nurse 6

Participants were happy that the functionality to switch between imperial and metric had been maintained from the web-based version of MUST. Positive comments were also made about the automatic calculation function meaning that there was less chance of error.

#### Wording and Misinterpretation

Feedback about wording was generally positive, although, there was concern around the use of the word malnutrition and how this might be perceived by patients:

My only concern is around the wording and with malnutrition, and it's just something that we've had where we've had to explain, you know, a letter that's gone to a GP about risk of malnutrition and it's copied to a patient

Dietitian6

Participants also felt that the wording could be clearer in the introduction and there were also concerns about the use of the word “sex” over “gender.”

### Barriers and Facilitators in Different Settings

#### Suitability in the Community

Many of the participants felt that the app would be particularly useful for GPs and HCPs working in the community. It was also thought that care home staff would find the tool valuable for routine screening:

I do a lot of training with care homes around how to identify malnutrition and we use MUST, you know I teach them how to do MUST scores a lot and I think an app would be really useful for them.

Dietitian4

Participants also commented on the inaccurate MUST scores that are referred to them and believed that the MUST app would help by preventing calculation errors:

…in care homes, we would get a lot more accurate referrals through rather than people calculating using paper-based version.

CDt2

However, it was also mentioned that those using the app should be offered training on how to use it.

#### Suitability for Patients and the General Population

There were mixed opinions about the target audience for the app with some considering that patients and the general population may not know the information required for completing MUST:

…its asking a lot of information that we as healthcare professionals can get. But asking somebody how much weight they’ve lost in the last 3-6 months; the likelihood is that they’re not going to know.

CDt2

It was also thought that the results screen was targeted at HCPs and that this would not be appropriate for patients. It was also stated that there may be difficulties with downloading the app or even issues created if members of the public panicked after seeing a high or red score. In contrast, others thought it important to self-screen and highlight the risk. One suggested having a poster with a QR code to provide easy access to the app for self-screening. It was also thought that as other MUST tools are freely available then the app should be the same. Although, it was also highlighted that use by the general population may lead to inaccuracies and as MUST require training it probably would not be suitable for patients to use, and if patients did use it then the app may need adapting with additional instructions. Finally, it was suggested that the app could be open to everyone if it was kept clear and simple with signposting for both HCPs and patients.

#### Suitability for Hospitals

Participants were very complimentary of the app, and all gave their approval for its use in health care, with many stating that it would be a useful tool for improving routine screening. All participants agreed that the app was far better than the paper-based tool and some also stated that they preferred the app to the web-based version. Although, one participant highlighted that there are already alternative tools available. Most participants were keen to see the app adapted for their particular trust or electronic systems or to be able to store profile data and results. However, it was also thought that information on local policies could be kept simple:

It does say on the management guidance for each of the MUST levels to follow local policy, so it could be that when you have your own Trusts policy it’s probably more around educating within the Trusts.

Dietitian12

It was also thought by one participant that the app results page should be kept as generic as possible as the next steps in a patient’s care pathway could differ greatly between patients with numerous possible treatment options.

## Discussion

### Principal Findings

This was the first exploration of user views on the newly developed MUST mobile health app, which identifies people at risk of malnutrition. Overall feedback for the app was extremely positive. Many of the participants agreed that the app would be a useful tool to improve routine screening of malnutrition risk, particularly in community settings, which has been highlighted as a gap in practice [28]. Previous work has demonstrated that there are several barriers to malnutrition screening in the community, including short general practitioner (GP) appointment times, overwhelming workloads [29], HCPs doubting the value of screening [30], poor recording of patient’s weight in medical records [29], and uncertainty about roles and responsibilities pertaining to screening amongst HCPs [31,32]. The MUST app may be able to address some of these issues by presenting the tool in an easy, accessible form for use in different health care settings.

For the app to work in practice it is essential to provide users with an interface that is inviting and intuitive to use. Focus group participants provided feedback on how the app could be improved or changed to make it more user-friendly or accessible to all types of users in different health care settings. The demographics screen was seen as a big barrier to the implementation of the app by deterring people before they even started to engage with the screening tool. Incentives for completion of the demographics, such as auto-filling electronic records, or at least linking to electronic records may help to mitigate this issue and may be a reality in the future with the NHS digitalization vision [33]. However, the feedback about demographics suggested that it may still be important, if not necessary, to have the option to skip these questions altogether to encourage use. The demographics screen will be essential for the initial trialing and testing of the app in practice; therefore, it may be helpful to add extra information at the start of the app to clarify why demographics are necessary and manage expectations around the purpose of demographics, emphasizing that results will be generic and not tailored to any information provided.

There was a lot of agreement across the focus groups that the calculation of the MUST score can be confusing or misunderstood. Participants reported that the same mistakes are made repeatedly by different groups of HCPs. This agrees with a previous study, which audited screening in one hospital and reported that MUST scores were calculated incorrectly as BMI was miscalculated or previous weight from 6 months ago was unknown [34]. The automatic calculation provided by the MUST app will go some way to improve these discrepancies. However, if we also endeavor to apply the extra information and options as suggested by these focus groups, such as allowing for edema, then the MUST app may be able to provide more clarity and help users to record MUST more accurately. Most participants were also keen to see links out to the MUST explanatory booklet [35] and highlighted that the information contained in the booklet is essential for the correct implementation and calculation of MUST. From the feedback, it was clear that the correct use of the app would be greatly improved by the addition of alternative or subjective measures, due to the difficulties in obtaining weight and height information. However, it would be essential to provide clear guidance around the use of alternative or subjective measures and how they are to be taken. In addition, as there are clear guidance about alternative and subjective measures within the MUST explanatory booklet [35] it may be more appropriate to continue to signpost to this rather than to incorporate this measurement information into the app. Although, the ability to record these types of measures in the app should be considered as it would provide increased opportunity for HCPs to complete screening.

In terms of sustainability and integration, the MUST tool itself was considered by most participants to be a “crude” tool that often-missed at-risk patients or prevented the flagging of certain at-risk patients. As the MUST app is a copy of the original tool, these issues will be reflected in the app. One of the major points to come out of the integration discussion was that electronic versions of MUST are already built into electronic systems within hospitals and some GP surgeries. Therefore, it is likely that the MUST app will be of more use in community settings where there is a lack of access to an electronic version of MUST. As such it would be prudent to view the app as a tool to be used in primary care and in the community and further developments should be in line with this. Having easier access to the MUST tool in all health care settings fits in with a recent recommendation to have standardized use of one preferred malnutrition screening tool per health care setting, so to facilitate the implementation of routine malnutrition risk screening [15]. The use of the app in GP settings could present a worthwhile opportunity for encouraging GPs to screen for malnutrition risk by the provision of an easy-to-use and accessible screening method that would aid in maintaining up-to-date records on patients’ weight and BMI. In addition, using the MUST app in the community will also provide an excellent opportunity for validation of MUST in the community setting, as even though MUST has been substantially validated in hospital and care-home settings [1,36,37], validation studies of MUST are lacking in community-dwelling older adults [18]. Funding and support for the app long term took the focus group discussions back to using the app in secondary care with suggestions of integration into the BAPEN national audit system [13], integration into health care handheld devices, and integration into NICE malnutrition guidelines [38]. As MUST is already the most commonly used tool in hospitals [11], it would be reasonable to build on this by using the app in these directive-driven areas of secondary care. Therefore, to allow the app to be sustainable it may be crucial to use the app for supporting national and local targets in the hospital setting as well as providing a functional and useful tool for community care.

Participants in the focus group had a very positive response to the design, functionality, and useability of the app, stating that the dropdown boxes and automatic calculation made it quick and easy to use. This suggests that the app would be a great option for busy HCPs as the duration of screening time and the burden on HCPs’ time has been highlighted as a barrier to completing screening [30]. The positive feedback relating to user experience design also makes this app a good prototype and a useful version to take forward to the next phase of development. Although, the focus groups did provide some useful information on how the app’s appearance, functionality, and descriptions could be improved to provide a better understanding of the app and a better user experience.

Feedback about the suitability of the app in the health care system was very positive and it was thought that community HCPs and care home staff would find the tool extremely useful. However, feedback also suggested that before the app is released, it may be appropriate to consider offering training to potential users or finding out if current MUST training could be used for additional training on the use of the app. This would also bring the app in line with the current NICE recommendation of “screening for malnutrition risk being carried out by HCPs with appropriate skills and training” [38]. In terms of the target audience and who could use the app, the overall feedback was very mixed; however, it was clear that the app would need further development to be suitable for use by the general population, despite self-screening being highlighted as a major benefit. It was also noted that use by the general population may lead to inaccuracies and as the app has the potential to be used as a national monitoring system for malnutrition risk, this would not be appropriate. This work has also highlighted the barriers and facilitators for the use of the app in different settings, with barriers for use with patients and the public far outweighing the facilitators.

### Conclusions

The MUST app provides an easy and accessible way to screen for malnutrition risk, particularly in community settings. It was positively valued by potential users and considered highly useful for improving the routine and accuracy of screening. Most participants considered the app to be for professional use only, stating that patients may find it too technical or too clinical. There was also concern about the app causing unnecessary panic and misuse of the app by patients, leading to inaccuracies. Participants also made suggestions for app sustainability and improvements, such as the addition of more subjective measurements and tips on how to measure ulna length. Future work would include trialing the app as well as considering further development for potentially mapping and monitoring malnutrition risk across the United Kingdom. It would be appropriate to conduct further evaluation with community-based practitioners, with any feasibility or pilot studies focusing on health care settings within the community.

## Appendix

Multimedia Appendix 1 COREQ (Consolidated criteria for reporting qualitative studies) checklist.

Multimedia Appendix 2 Focus group topic guide for the evaluation of a mobile app for the Malnutrition Universal Screening Tool (MUST).

Multimedia Appendix 3 Thematic framework.

Multimedia Appendix 4 Relevant quotations for each subtheme.

## Abbreviations

ADE: acute disease effect
BAPEN: British Association for Parenteral and Enteral Nutrition
CDt: community dietitians
COREQ: Consolidated Criteria for Reporting Qualitative Research
GP: general practitioner
HCP: health care professional
MUST: Malnutrition Universal Screening Tool
NICE: The National Institute for Health and Care Excellence
NHS: National Health Service

## Data Availability

The datasets generated and analyzed during this study are available from the corresponding author on reasonable request.
