# Supplementary material for: Exploring Individuals’ Views and Feedback on a Nutritional Screening Mobile App: Qualitative Focus Group Study
Source: JMIR Form Res. 2024 Dec 18;8:e63680. doi: 10.2196/63680 (PMC11694050; doi:10.2196/63680)
Supplement: Multimedia Appendix 2 [file formative_v8i1e63680_app2.docx]

**Welcome**

Welcome to our focus group, it’s great to see you all here today and thank you so much for joining us. My name is <Researcher> and I’m a researcher from the University of Manchester and I’ll be leading us on this discussion today.

Joining us on the call we have <Researcher> and she’s here today to help support me and the group in general and make sure we stay on topic and stick to time.

Before we begin I’d just like to provide a brief overview of the app and our discussion here today. I’ll also go over some ground rules and reiterate the confidentiality of this focus group.

**Overview**

This focus group will be an organised discussion with you as a group to understand your views and opinions on the newly developed mobile application for MUST.

MUST, short for Malnutrition Universal Screening Tool, is a tool that is used to assess a person’s nutritional status in healthcare settings. Currently, MUST is only available as an online tool or as a paper based version. In this focus group today we will look at the newly developed app version of this tool and evaluate its content, functionality and usability.

We hope to use this focus group to capture useful information from yourselves, which would not be as detailed if we were using questionnaires. The group will be open and collaborative so that you may build upon each other’s responses.

**Recording of the focus group**

Please be aware that when we start the focus group in a moment I will start recording the session and this will include a recording of your face and voice. If you prefer, you can disable your camera so that only your voice is recorded. I will let you know when recording starts and if you wish to do so you may disable your camera at this point. Recordings will be later transcribed anonymously and then deleted.

**Confidentiality**

Please see your participant information sheet for full details relating to confidentiality. But also, please be aware that all information collected will be confidential and participants’ names will not be disclosed, however, short quotations may be used in writing up the findings. As is usual with focus groups, we ask you to keep confidential what is said in the group. I hope this encourages you to speak openly.

**Ground rules**

If it seems that we are getting off the focus, I might just try to move the discussion on so that we stick to time. It is useful as well to have a few ‘ground rules’:

- As we will be recording the group, it would be helpful if people could speak clearly and one at a time. I will try to go around the group and ask you for your responses in order.
- Everybody will have an opportunity to speak and listen.
- There are no right or wrong answers, we want to find out about the views and opinions of everybody, and people will have different views and opinions.
- Everybody’s views and opinions are important to us.
- You can decide what you want to share, and do not want to share. You don’t have to talk about anything if you don’t want to.
- Everything that is said in the group should stay in the group, that is; it is confidential.
- Does anybody have any questions? Are you happy for me to start recording?

**Let’s begin**

I’d like to find out some more about you by going around the group one at a time. Please tell me your first name, (job role if HCP), and any experience you have with malnutrition or MUST.

Great, thanks so much for sharing.

Has everybody managed to download the app? I will now run a short demonstration of the app.

------DEMO OF APP------

**Questions**

1. Firstly, have you had opportunity to download the app to your phone?
2. What are your first impressions of the app?
3. What do you think of the layout?
4. How can the layout be improved?
5. What do you think of the colours and branding? Would you change anything related to colours, branding or style?
6. What do you think about the functionality (ease of use)?
7. What would you change, if anything, to make it easier to use?
8. What do you think about the layout of the results at the end and
   1. is this useful and easy to understand?
9. How does the app compare to the paper based and online versions.
10. Is there anything missing that the paper based and online versions have?
11. What else you would like to see included in the app?
12. Can you see any potential problems about the app working in practise?
13. Should the app just be available to HCPs or would it be useful for patients to have access for self-assessment?
14. How should we make the app sustainable? (For example: Pay per download, advertising or a combination)
15. Is there anything else you would like to tell us?

**Close**

Fantastic, that was so useful. I’ll now stop the recording. Thank you so much for joining us today.
