# Supplementary material for: Exploring Individuals’ Views and Feedback on a Nutritional Screening Mobile App: Qualitative Focus Group Study
Source: JMIR Form Res. 2024 Dec 18;8:e63680. doi: 10.2196/63680 (PMC11694050; doi:10.2196/63680)
Supplement: Multimedia Appendix 3 [file formative_v8i1e63680_app3.docx]

| Index | Description |
| --- | --- |
| 1. **Improving MUST app for better use in practice** | |
| 1.1 Demographics screen | Feedback and suggested changes for the demographic questions |
| 1.2 MUST calculator | How the calculation score can be improved or adapted to account for individual patients and how MUST score is affected |
| 1.3 Results and signposting | Feedback for how results are displayed and suggestions for signposting to further information |
| 1.4 Sustainability | Ideas and suggestions for keeping the app up to date and accessible over the long term |
| 1. **User Experience Design** | |
| 2.1 Colours, design and functionality | Feedback on app branding, layout, functionality and if it was considered user-friendly |
| 2.2 Wording and misinterpretations | Suggestions for changes to wording and concerns over how some things might be misinterpreted |
| 1. **Barriers and facilitators in different settings** | |
| 3.1 Suitability in hospitals | Suitability of the app for use in hospitals and its integration into existing systems |
| 3.2 Suitability in the community | Suitability of the app to be used in community settings such as care homes and General practitioner surgeries |
| 3.3 Suitability for patients and general population | Suitability of the app to be used by non-healthcare professionals such as patients, carers and members of the public |
