# Supplementary material for: Exploring Individuals’ Views and Feedback on a Nutritional Screening Mobile App: Qualitative Focus Group Study
Source: JMIR Form Res. 2024 Dec 18;8:e63680. doi: 10.2196/63680 (PMC11694050; doi:10.2196/63680)
Supplement: Multimedia Appendix 4 [file formative_v8i1e63680_app4.docx]

| Theme | Quotes |
| --- | --- |
| **1. Improving MUST app for better use in practice** | |
| 1.1 Demographics screen | ‘*people who self-screen are going to open it and go ooh wait a sec I’m suddenly getting asked all these questions about postcode and demographics and actually…I don’t know. I was just wondering if it would make more sense to go straight into the questions height, weight, MUST and then the demographics afterwards*’ (Dt11).  ‘*people, particularly patients and unpaid carers and family member might be thinking “ooooooh” because some people are concerned about data gathering and why is that required. So, in terms of ease of use I can see that potentially being off putting*’ (Dt13).  ‘*I think the big question it’s about convenience and speed. Is it quick to use. Because the whole point of MUST is that it is, you know the questions are basic to get the answer. And if people are going to have to put in more data than they have time for then they may not want to use it*’ (Dt7).  ‘*maybe if there is a pre selection thing, this is my setting, I want to collect everything as though it’s a medical record, or I just want to do a MUST calculation*’ (Edu1).  ‘*I’m looking at the diagnosis and imagine I was a patient and I’m not sure what disease I’ve got. So I’m not sure whether its appropriate for patients or if an option like ‘don’t know’ needs adding*’ (NN4).  ‘*My feeling on the diagnosis stuff as well was, I mean I understand you’re just doing it for research purposes, but when I was filling it in I was thinking oh are they going to give me some advice because I’ve got a frail patient*’ (Edu4).  ‘*The postcode, are you expecting the patients home postcode or their location. I can see why you want both, but I think it would be important on the app to say current location or patients home postcode*’ (NN3). |
| 1.2 MUST calculator | ‘*could you have a drop-down box to say whether it's estimated, actual or MAC*’ (CDt1)  ‘*Does it also give the opportunity to record whether it’s a self-reported weight or an actual weight*’ (Dt15)  ‘*actually state how the weight was taken. So hoist, standing still, seated scale*’ (Dt17).  ‘*weight 3-6 months ago, does there not need to be a box to say ‘don’t know’ because in my experience that’s the one thing that’s never filled out properly in our hospital*’ (Dt11).  ‘*the ADE score, I wouldn’t have that on an info button you have to click on, really that needs to be there because really that’s one of the things that isn’t understood*’ (Dt4).  ‘*my understanding is, its if you are acutely unwell and if you have had or unlikely to have little or no intake over the last 5 days. So my suggestion would be to highlight or bold the word ‘and’. This is where things go wrong in my experience*’ (Dt3).  ‘*is there any information on there around amputation and oedema and how you account for those?*’ (Dt15).  ‘*sometimes we use the mid upper arm circumference or subjective evidence of weight loss*’ (Dt5).  ‘*looking at the mid arm circumference as a potential surrogate measure for BMI, especially if you’re trying to think about how this can be used in primary care*’ (Dt1).  ‘*Its including basic information like height and weight and weight history. So I suppose its just what if somebody, if you're picking it up and you didn't have all that information?*’ (Dt5).  ‘*if you want people to use ulna height you need to have a link to show people how to measure ulna height*’ (Dt8).  ‘*But if you do decide to include them [how to measure ulna and mid arm circumference] then I would suggest that you still include the little pictures in there to show how to take the measurement*’ (Dt9).  ‘*my understanding is, its if you are acutely unwell and if you have had or unlikely to have little or no intake over the last 5 days. So my suggestion would be to highlight or bold the word ‘and’. This is where things go wrong in my experience*’ (Dt3).  ‘*I know when we’ve been developing our online system here we’ve had to stipulate that we can’t call it a MUST score if we’re using a MUAC because its subjective and so we can’t class it as an actual MUST score. Because BAPEN won’t endorse it in that sense. So it’s a very funny and grey area when you’re trying to develop it and whether BAPEN approve it or not*’ (NN1). |
| 1.3 Results and signposting | ‘*can we see what you’ve done, what information you’ve actually put in. If there’s a functionality where you can see what you’ve entered*’ (Edu2).  ‘*I know at the bottom you signpost to the booklet. Could there be a link, I’m just thinking in practise where can people find information*’ (Edu2)  ‘*maybe give people options to go to other places for information on…..because your not necessarily going to be a dietitian that’s doing it*’ (Edu4).  ‘*Theres pictures in the MUST booklet aren’t there*’ (Dt5) |
| 1.4 Sustainability | ‘*where it says with a MUST of 2 refer to the dietitian, that certainly in some hospital settings there just aren’t enough…….basically the dietitians weren’t necessarily set for referrals with a MUST of 2’* (Dt3).  ‘*I think the other issues are around MUST not picking up every patient, you know based on them having dysphagia and things affecting their ability to eat is probably a MUST issue rather than and app issue*’ (Dt12).  ‘*a lot of patients we would like to flag wouldn’t flag on the acute disease effect step, particularly with it just being either you just score a 2 or you don’t score at all*’ (NN6).  ‘*If a nurse fills in a weight then weight is automatically pulled through and put into the electronic MUST and MUST updated. So there’s lots of ways things talk to each other so I don’t know then in my Trust if they would want to use a separate app to record MUST that wasn’t talking to the other bits of that patients care*’ (Dt11).  ‘*we’ve just launched this new system called [electronic system name] and it would just be the difficultly of flicking between the two systems*’ (Dt14)  ‘*we have it built into our computer system so we would probably use that to do our MUST screening*’ (Dt16).  ‘*our MUST entered an electronic version, its pretty clunky and clumsy but we’ve done the best job we could in trying to make it nice and easy, for example you put in your ulna length and it spits out your height*’ (Dt9).  ‘*In the future, is there going to be a user licence agreement. As in, this is our Greater Manchester high-risk care plan. That could be a potential way to income generate for BAPEN really. Or thinking about how does this link into primary care? How is going to make the life of the busy GP actually bother to think about nutrition screening when someone is in front of them*?’ (Dt1).  ‘*But if there was a proper incentive nationally from BAPEN or other groups to say its really important this data is not just used at Trust level but its feeding into somewhere else, then I think that would definitely help*’ (Dt11)  ‘*I would have thought BAPEN’s got an invested interest because they have the equivalent on their website and that’s what we use in the national audit, or we download you know the audit online questionnaire. I always found it a little bit confusing, but I would think that its another method that BAPEN can have access to national data*’ (Dt17).  ‘*a way to negotiate with BAPEN about using this and being able to update it regularly is that certainly from my perspective this would be an app that I would recommend to download to the handheld devices or even the PCs at my trust because we’re fully EPR. Because our EPR system, without wishing to sound unprofessional is quite tricky to navigate, certainly from a nutritional screening point of view, so this would be really, certainly the way I’ve used it, really quick and easy to get it done and it would give colleagues support. So therefore, you could approach the Trusts to have a small charge to download it to the devices and then that’s a way of additionally funding it. Does that makes sense. If you engage with the core groups of BAPEN I can’t see any of the core groups not agreeing to support this and enter into the trials. Because I think this is really good and there’s lots of our NNNG members, they’re not just nutrition specialists, they are nurses in care homes, they are community nurses district nurses. So is something they could use as well, so it has lots of potential out there. And so one way is getting money from Trusts to download it to their handheld devices. Because I know district nurses have handheld devices as well*’ (NN3)  ‘*perhaps speak to NICE about linking it into the NICE guideline as obviously NICE does suggest people have a MUST score. Whether or not they’d give you any funding I don’t know but it might be a way of getting it out there more*’ (Edu4)  ‘*I’ve never been able to achieve that nice guideline recommendation that every outpatient that comes to our hospital is screened by a MUST because it’s not possible for the staff in every clinic of the hospital to do it. So, some way to link it to that standard’* (Dt16). |
| **2. User Experience Design** | |
| 2.1 Colours, design and functionality | ‘*First impressions are that it looks really clear, nice colouring, nice and bold and fresh*’ (Dt7).  ‘*I think it all looks good, you know easily recognisable as the MUST tool that we all know, it’s got the BAPEN logo its colour coded as it would be online and on the paper version*’ (Dt12).  ‘*I like the fact that it was colour highlighted for the low, medium and high*’ (NN1)  ‘*there’s no faff around it. It’s very straight to the point which I like*’ (CDt2).  ‘*So, if you were doing MUST in clinical practice, you would expect it to come in that order and you’d probably have that information ready to input. And the boxes came up in that order so yeah that was great*’ (Dt12).  ‘*having the terminology being similar, so step 1 is BMI and step 2 is weight loss. That might be helpful*’ (Edu1).  ‘*especially the box where it’s all laid out, it shows somebody how the scores been calculated*’ (Dt4)  ‘*very self-explanatory, very straight forward. I like the fact that it has dropdown choice boxes, so it’s quite quick to do rather than having to input all the information sort of by hand almost. From the point of view of busy nursing staff that’s good*’ (NN6).  ‘*I like the fact you can switch between imperial and metric as well. A lot of our elderly patients work in stones, and we don’t always have to hand what the equivalent is in new money*’ (Dt13).  ‘*I think it was less room for error with the calculations. So, I think that’s really good that it can calculate for you*’ (Dt6) |
| 2.2 Wording and misinterpretations | ‘*I like the way its colour coded, not too wordy either. You know your instructions of what to do next*’ (CDt2)  ‘*My only concern is around the wording and with malnutrition, and it's just something that we've had where we've had to explain, you know, a letter that's gone to a GP about risk of malnutrition and it's copied to a patient*’ (Dt6).  ‘*where it says tell us a little about yourself or client. I found that confusing, is it collecting my info as a user profile or is it for the person beings screened. So just the wording on the intro I found a little confusing*’ (Edu1)  ‘*One is about you say select sex and I just wonder whether sex or might gender be a bit gentler. And I know there’s a binary thing about male and female and I know we using MUST and what that represents. But sometimes its not quite that straight forward*’ (Edu2). |
| **3. Barriers and facilitators in different settings** | |
| 3.1 Suitability in the community | ‘*The other thing is this could be really great for GPs, practice nurse, you know wider people out in the community within MDT community health teams*’ (Dt15).  ‘*in care homes, we would get a lot more accurate referrals through rather than people calculating using paper-based version*’ (CDt2).  ‘*I do a lot of training with care homes around how to identify malnutrition and we use MUST, you know I teach them how to do MUST scores a lot and I think an app would be really useful for them*’ (Dt4).  ‘*I think the important thing is that it’s appropriate for people and that they have access to some training on how to use it*’ (Dt5).  ‘*if you’re in the community, maybe where you don’t have access to notes where you’re able to complete remotely, being able to actually store several of them [MUST score entries and results] on your device. So that when you get back to base camp, wherever that may be, you’re able to actually go through….well this was patient 1, patient 2, patient 3 and I can input them. Rather than having to make a note of it elsewhere outside of the app*’ (Edu1). |
| 3.2 Suitability for patients and general population | ‘*its asking a lot of information that we as healthcare professionals can get. But asking somebody how much weight they’ve lost in the last 3-6 months, the likelihood is that they’re not going to know*’ (CDt2)  ‘*I’ve just put it in to come up has high risk and how its reading is very much the advice that healthcare professionals should follow…..So if a patient did this and got a score of 4 and it says implement local policy, set goals that’s probably not….its more you need to sign post them to other things or contact GP or something*’ (Dt11).  ‘*particular patient groups may not have the skills to download and use the app*’ (Dt8)  ‘*But if available to all then GPs may get inundated with people scoring MUST 2 or 3. If patients see a big red box it might cause panic*’ (Edu1).  ‘*Personally, I do think that we should be promoting self-screening and highlighting risk. I know within certainly within GM when we were doing nutrition hydration work with things like the paper weight armband that was very much our aim was to highlight to the general public the risk of malnutrition*’ (Dt1).  ‘*I’ve been reviewing our malnutrition screening tool completion and I think it would be really handy to have a QR code on a poster in places where they can just click onto it and it goes over to the app. People can then just self-screen*’ (Dt10).  ‘*they are all freely available. And I suppose no-one would come across it if they weren’t seeking out to do it and I think if they’re looking for MUST they must have seen an information leaflet or had advice from a professional’*  (Dt12).  ‘*I think its better to just have it for HCPs if you want it to be accurate*’ (Dt4)  ‘*Definitely healthcare professionals, because there's definitely an element of training here around the tool, isn't there? And I'm not quite sure about patients using it*’ (Dt6),  ‘*I would say on that in terms of the height and weight if the patients doing it do you want to have instructions in terms of taking shoes off, heavy jewellery, just kind of simple things that people might not be aware of that will influence their height and weight*’ (Edu5).  ‘*but also I think its looking at what we want to achieve with this app? So its not going to be detailed medical advice, so if we keep it relatively simple and it is just a signposting thing I can’t see why you necessarily need to split it its just making it clear: If you are a patients and your curious…..You click on this link, if you are a healthcare professional you click on this link or go here on your intranet*’ (NN3)  ‘*I think it would be really nice to have a proper app available that we know is validated and makes sure people get to the right MUST score would be really helpful*’ (Dt4). |
| 3.3 Suitability for hospitals | ‘*If wanted to do an audit and just get students to do an audit for the day and we need the results the next day for a meeting it would be nice to think that we could export it somehow*’ (Dt17).  ‘*have some way of monitoring someone’s weight and MUST score overtime so there was a page to say last month this was what your score was, last year this was what your score was*’ (Dt9).  ‘*You know, you could put your own stuff on there, you know can it be linked with other systems. Can you drop this app into another software*’ (CDt1).  ‘*It does say on the management guidance for each of the MUST levels to follow local policy, so it could be that when you have your own Trusts policy it’s probably more around educating within the Trusts*’ (Dt12).  ‘*In an acute setting you’re not always able to do that and you might need to start somebody on sip feeds. You’re then obviously getting the app to work in different locations and settings. So trying to make it as generic as possible is important*’ (Dt4)  ‘*I think it’s more user friendly overall than the online tool that’s available on BAPEN*’ (Dt3).  *‘I mean I’ll be honest with you I’m a huge fan of the R map tool, which is another one and I’m also a fan of the self-screening tool. But this is good….this is far better than anything paper’* (NN2) |
